# Co-design of interventions and services with structurally marginalized populations in the context of maternal and early childhood primary care: a rapid scoping review

**DOI:** 10.1017/S146342362510011X

**Published:** 2025-06-16

**Authors:** Lucie Vicat-Blanc, Lisa Merry, Marie-Christine Harguindéguy-Lincourt, Yujia Tang, Andraea Van Hulst

**Affiliations:** 1 Ingram School of Nursing, Faculty of Medicine and Health Sciences, McGill University, Québec, Canada; 2 Faculté des sciences infirmières, Université de Montréal, Québec, Canada; 3 La Maison Bleue, Montréal, Québec, Canada

**Keywords:** co-design, early childhood, marginalization, maternal child health, social isolation, primary care

## Abstract

**Aim:**

To synthesize evidence on approaches used in the co-design of maternal and early childhood primary care interventions with structurally marginalized populations.

**Background:**

Involving end-users when developing health interventions can enhance outcomes. There is limited knowledge on how to effectively engage structurally marginalized populations (i.e., groups that are affected by structural inequities resulting in a disproportionate burden of social exclusion and poor health) when co-designing maternal child primary care interventions.

**Methods:**

A rapid scoping review was conducted by searching EMBASE and CINAHL for studies indexed between January 2010 and December 2024. Peer-reviewed studies describing co-designed health interventions or services tailored to structurally marginalized populations during prenatal, postpartum, or early childhood periods were included if they reported on one or multiple steps of a co-design process in community-based primary care practices in high-income countries.

**Findings:**

Of the 5970 records that were screened, nine studies met the inclusion criteria. The co-designed interventions included three eHealth tools, a health- and social-care hub, a mental health service, a health literacy program, an antenatal care uptake intervention, an inventory of parenting support strategies, and a fetal alcohol spectrum disorder prevention campaign. Women, mothers, fathers, and health- and social-service providers contributed to the co-design process by participating in workshops, focus groups, individual interviews, or surveys. They provided feedback on intervention prototypes, existing resources, and new intervention designs or practice models. Ethical and practical considerations related to the population and context (e.g., marginalization) were not consistently addressed.

**Conclusion:**

This synthesis on intervention co-design approaches with structurally marginalized populations can provide guidance for primary care organizations that are considering maternal child health intervention co-design with this clientele. Future work should include a critical reflection on the ethical and practical considerations for co-design with structurally marginalized populations in the context of maternal and early child care.

## Background

Prenatal, postpartum, and early childhood periods are critical for the provision of preventive and health promotion care, which lay the foundation for the rest of the child’s life (Gadson *et al*., 2017; Nussey *et al*., 2020; Reyes *et al*., 2021; Webb *et al.*, 2017). Structurally marginalized populations may face difficulties in obtaining timely and high-quality maternal child health care (Samb *et al*., 2019) and are less likely to access available care due to factors such as low financial means, linguistic barriers, low health literacy, lack of culturally safe care, or previous negative interactions with the health care system (Baah *et al.*, 2019; Loignon *et al*., 2022). Structural marginalization arises from inequities embedded in policies, practices, beliefs, and values that limit choices and opportunities while increasing exposure to risks, harms, and adverse health outcomes (Browne *et al*., 2012; Powell, 2013). Examples of structurally marginalized populations include but are not limited to people with low socioeconomic status, racialized people, Indigenous people, and people with precarious migrant/refugee status. While structurally marginalized populations often face difficulties in obtaining maternal child primary care, they are also well positioned to provide invaluable input when designing health care and social services meant to tackle these same challenges (Mulvale *et al*., 2016; Samb *et al.,* 2019; Rogers *et al.,* 2020).

Co-design in healthcare is a collaborative approach that emphasizes the equal partnership of three key players, namely healthcare providers, patients and their families, and decision makers, in the development of health services and interventions in order to better meet the needs of care recipients (Jessup *et al.,* 2018; Ward *et al*., 2018). Co-design has been proposed as an approach to reduce maternal child health disparities by providing a better understanding of cultural and socioeconomic factors, and health beliefs and practices, all of which differ from one person to the next and may impact access to care and individuals’ responsiveness during service provision, particularly among structurally marginalized populations (Mulvale *et al.*, 2016; Rogers *et al*., 2020).

Two review studies by Rogers *et al.* (2020) and Samb *et al*. (2019) provided an in-depth analysis of the different models of care associated with improved health access among structurally marginalized populations. Both studies highlighted the need for more involvement of the targeted clientele in the development of health interventions to ensure that services are tailored to the specific needs of targeted populations. Rogers *et al.* (2020) suggested using co-design to provide services that are most responsive to the specificities of the communities. More recently, two review studies focused specifically on co-design approaches with structurally marginalized populations. A systematic review by King *et al.* (2022) described the state of knowledge on co-designed health interventions and services for Indigenous children and youth and reported that, although becoming increasingly popular, there is a lack of understanding on the co-design process for this clientele and on the extent to which co-design can help to achieve equity (King *et al.*, 2022). Another systematic review by Rustage *et al*. (2021) analyzed the different participatory research approaches used to co-develop health interventions with migrants and concluded that to optimize co-design with migrant populations, it is essential to address their socio-cultural needs as well as potential power imbalances. Although these two systematic review studies provide some evidence on co-design approaches with structurally marginalized groups, no review focused specifically on co-designing health interventions and services in the context of maternal and early childhood primary care. There is a need for synthesized information on how to co-design such interventions (e.g., who is involved, what happens during the process) in order to support maternal child health organizations interested in implementing co-design approaches with structurally marginalized populations.

This project was conducted through a longstanding partnership between a School of Nursing and La Maison Bleue, a non-profit organization that provides interdisciplinary and integrated health, social, and psychoeducational services during pregnancy and early childhood to individuals and families facing vulnerable contexts via a unique network of community-based clinics across Montréal, Canada (Aubé *et al.*, 2019). One of La Maison Bleue’s current priorities is to engage and consult with their clientele to further improve services and better respond to their needs. To support La Maison Bleue towards this goal, we conducted a study to synthesize evidence on co-design processes with structurally marginalized populations in the context of maternal and early childhood primary care. Specifically, we sought to answer:

1. Who is involved in the co-design process?
2. How and when do structurally marginalized populations participate in the process?
3. Which strategies are used to reach and engage structurally marginalized populations?
4. What are practical and ethical considerations when co-designing health interventions with structurally marginalized populations?

## Methods

A rapid scoping review was conducted according to the Joanna Briggs Institute (JBI) methodology for scoping reviews and the Arksey and O’Malley (2005) framework (Peters *et al.,* 2015, 2020). The preferred Reporting Items for Systematic Reviews and Meta-Analysis-Scoping Review (PRISMA-ScR) reporting guidelines were used (Supplementary File 1). Rapid scoping reviews combine methodologies of the scoping review with those of rapid reviews (Dobbins, 2017; Bouck *et al.*, 2022; Garritty *et al.*, 2020). They are exploratory and descriptive in nature and involve a comprehensive search of the literature, but are limited to fewer search databases and rely on a single reviewer (Peters *et al.*, 2020). This methodology was selected for feasibility reasons so as to provide the partner organization with synthesized knowledge within a timely fashion and inform local co-design practices. Members from La Maison Bleue (including co-author MCHL) were involved in formulating the research question and provided extensive feedback on the review protocol, results, and recommendations.

### Search strategy

An initial exploratory search of Embase (OvidSP) and CINAHL (EBSCO) was undertaken to identify articles on the topic. Relevant words from the titles and abstracts of initially identified articles and the index terms used to describe the articles were then used to guide the development of a full search strategy. The search was based on two key concepts, co-design and structurally marginalized populations; the studies were then manually searched to determine relevance to prenatal, postnatal, and early childhood (up to 5 years) periods. The search strategies, including all identified keywords and index terms, were verified and validated by an academic health sciences librarian before implementation. The searches were conducted on September 12, 2023, for articles published or indexed between January 2010 and September 2023, and were subsequently updated on December 20, 2024. The detailed search strategies are provided in Supplementary File 2.

### Inclusion and exclusion criteria

Inclusion and exclusion criteria are detailed in Table 1. Briefly, we included studies that mentioned the co-design, co-production, co-creation, or co-construction of health interventions, services, or programs tailored to structurally marginalized populations from the time of pregnancy until children reach the age of 5 years old, in the context of community-based primary health care practices (i.e., not specialty centers) in high-income countries. Studies were included if the authors provided information on the co-design process to contribute data to at least one of the study sub-questions. We included empirical studies using quantitative, qualitative, or mixed method designs, and published in a peer-reviewed scientific journal in English or French. Gray literature, abstracts, theses and dissertation, and conference proceedings were excluded. Systematic reviews were also excluded but their reference lists were examined for any potential articles that could meet the inclusion criteria. The reference lists of included studies were also searched for additional studies.

**Table 1.** Inclusion and exclusion criteria

Inclusion	Exclusion
Studies (quantitative, qualitative, mixed methods). Published or indexed in databases between January 1, 2010, and December 20, 2024	
Published in French or English	
Conducted in high-income countries as per the World Bank criteria	
Published in peer reviewed scientific journals	Gray literature
	Abstracts, theses, and dissertations or conference proceedings
Co-design with structurally marginalized populations (such as precarious financial status, low education levels, unwanted or adolescent pregnancy, social isolation, mental health or addiction problem, abuse or violence, single parenthood, precarious migration status, racialized people, and Indigenous people)	Does not identify the characteristics of structurally marginalized population sub-groups
Description of at least one step of the intervention/service co-design process	Only mention co-design with no explanation on the methods or processes used
Intervention/service targeting women, mothers, fathers, parents, families or children during the prenatal, postpartum, and/or early childhood period	Interventions targeting children exclusively above 5 years old
Community health centers	
Primary care	Secondary and tertiary care

### Study selection

Citations from all identified reports were imported to Covidence and duplicates were removed (Covidence Systematic Review Software, 2023). For the first screening step, titles and abstracts were assessed in Covidence for potential inclusion, and studies not meeting inclusion criteria were excluded. For the second screening step, full texts were assessed for inclusion by LVB and validated by AVH.

### Data extraction, analysis, and synthesis

Data were extracted from included studies and entered into a data extraction table by LVB and reviewed by AVH. The analysis of extracted data was both descriptive and qualitative in nature and sought to characterize the co-design process. Specifically, descriptive texts were grouped into categories, each corresponding to specific research questions (e.g., the different stakeholders and the phases in which they were involved; the strategies used to engage and include the participants when co-designing; and the types of contributions made by the participants). Lastly, the synthesis was also informed by the framework developed by Dietrich *et al*. (2017) on “vulnerable” user involvement in co-design. This framework provides information on approaches to consider when co-designing services, albeit non-health related, with structurally marginalized populations, namely: *resourcing*, which entails gaining insights into the problem to be addressed by looking for relevant input; *recruitment*, identifying relevant and a sufficient number of partner organizations and users for involvement; *sensitizing,* offering pre-co-design preparatory activities for participants; *facilitation,* providing guidance to participants during the co-design workshops; and *evaluation*, soliciting participants’ unique knowledge to assess the relevancy of the co-designed service.

## Results

**Figure 1.**
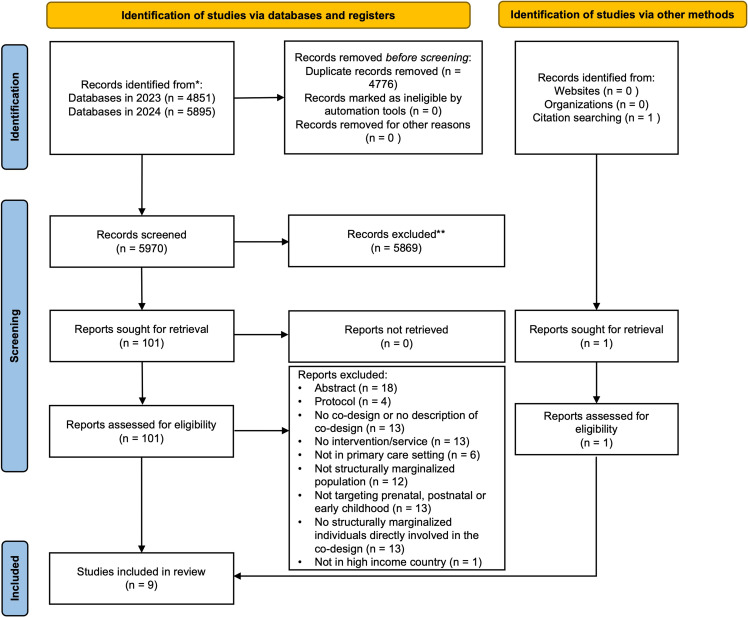
PRISMA flowchart for an overview of reports in co-design with structurally marginalized families.

Following duplicate removal, 5970 records were screened for eligibility. Nine studies met the inclusion criteria, of which one was identified through a reference list search of included studies (Figure 1). The most common reasons for exclusion were abstracts (e.g., conference proceedings) and studies with no direct involvement of individuals experiencing structural marginalization during prenatal, postnatal, or early childhood periods. Table 2 summarizes the characteristics of the included studies and describes the nine interventions and services being co-designed. Four interventions were co-designed for delivery during the prenatal and early postpartum periods, while the other five focused on the later postpartum and early childhood periods. The interventions included three eHealth tools (a breastfeeding support tool, a pregnancy, birth, and parenthood support tool, and a responsive feeding tool), a health- and social-care hub, a mental health service, a health literacy program, an antenatal care initiation intervention, an inventory of parenting support strategies for new parents, and a health campaign on prevention of fetal alcohol spectrum disorder.

**Table 2.** Characteristics of included studies and of the intervention co-design (*n*=9)

First author, year, country	Study aim	Intervention/service being co-designed	Description and number of community participants	Description and number of health-and social-care services providers
Abbass-Dick, 2021, Canada	To work collaboratively with young mothers in order to co-create an eHealth breastfeeding resource	A breastfeeding e-resource to meet the informational needs and help young mothers meet their breastfeeding goals	*N*=18 women under 26 y, expecting a child or had a child, had breastfed or were breastfeeding or planning to breastfeed, could read and speak English, had Internet access	*N*=8 health- and social-care services providers who worked with adolescents and young mothers in their communities to provide breastfeeding support
Baxter, 2024, Australia	To discuss and illustrate the application of a design thinking-led framework using mixed methods in the development of a health intervention with an underserved population group	Innovative digital child-feeding intervention supporting responsive feeding practices in families experiencing hardship and food insecurity	*N*=14 parents/caregivers of children aged 6 months to 3 years participating in a total of 3 workshops. *N*=29 parent/caregiver interviews *N*=213 parent/caregiver survey	*N*=9 child health experts participating in 1 workshop
Goodyear, 2022, Austria	To describe the development of service delivery approach for the “It takes a village” project that aims to identify and support children with parents experiencing mental illness	Service approach to assist adult health- and social-care services providers to identify parents of dependent children who may be seeking treatment for their mental health challenges.	*N*=2. No additional information.	*N*=37 health- and social-care services providers who may come into contact with the families with parents experiencing mental illness
Hall, 2023, Australia	To co-design, test and evaluate two integrated child and family hub models aiming to detect and respond to children aged 0–8 year experiencing adversity (e.g., socio-economic deprivation, maltreatment)	An integrated health- and social-care hub (i.e., a centralized service) providing intersectoral services and linkages to community-based support	*N*=2 parents of children ages 0–8 y with experiences of adversities and experience with services in the town, and available to attend all workshop	*N*=5 health- and social-care services providers who worked in local health, family services, community and early childhood services and health service delivery and strategic position within their organization
Montgomery, 2021, UK	To co-produce an e-resource to help prepare women who have experienced CSA (Childhood sexual assault) for pregnancy, birth and early parenthood	An e-resource that goes through the journey from deciding to have a baby to becoming a parent based on experiences of survivors of CSA	*N*=37 18y+, speak and read English and had been sexually abused in childhood and either had experience of pregnancy birth or were contemplating having a baby	*N*=unknown people with relevant research and professional expertise and the research team
Muscat, 2021, Australia	To revise and create a new version of a group-based health literacy program for new parents	A 4-weeks (4x2h) group-based health literacy program for new parents to better access, understand, appraise, and act on health information	*N*=3 new parents.	*N*=unknown number of health- and social-care services providers working directly with new parents, and managers of child and family health services”
Reid, 2022, Australia	To identify and refine culturally appropriate parenting goals support strategies for Aboriginal and Torres Strait Islander parents experiencing complex trauma	Identification of 8 culturally appropriate parenting goals and 60 related support strategies. (e.g., goal 5: “*practical assistance and life skills”* with associated support strategy “*assistance to reduce drug or alcohol use*”	*N*=37, parents and community members with an interest in Indigenous perinatal and maternal health	*N*=17, health- and social-care services providers (counsellors, psychologists) with an interest in Indigenous perinatal and maternal health
Sharma, 2023, United Kingdom	To develop and evaluate the acceptability and feasibility of a co-produced community-based intervention to increase uptake of antenatal care in an area with high ethnic diversity and low socio-economic status	Community out-reach using individual one-on-one and group sessions in public spaces and community organizations using a co-designed intervention script aimed to change behavior concerning early initiation of antenatal care	*N*= 20 local service users of different ethnic backgrounds	*N*=12 midwives, early years providers, young parent support workers, healthy lifestyle workers
Williams, 2024, Australia	To describes the development and implementation of the Strong Born Campaign from an Aboriginal context within Australia	A culturally informed health campaign (Strong Born) for the prevention of fetal alcohol spectrum disorder among Aboriginal people	*N*=6 Aboriginal women who played different roles in their families and communities participated in a focus group during co-design *N*= unknown, young Aboriginal women (young mothers and single women) in the community participated in a post-launch workshop	*N*=unknown experts in alcohol and drugs, disability, social and emotional wellbeing, child and maternal health, fetal alcohol spectrum disorder researchers, and cultural experts from across Australia

Studies published between 2021 and 2024, were conducted in four different countries: Australia (*n*=5), Austria (*n*=1), Canada (*n*=1), and the UK (*n*=2), were published in English. All nine studies mentioned the word “co-design” (or variation of the term, e.g., co-production or participatory design), however, only three provided a definition for the concept (Hall *et al.*, 2023; Baxter *et al.,* 2024; Sharma *et al.*, 2023) and four other studies mentioned using participatory research approaches (Abbass-Dick *et al.*, 2021; Goodyear *et al.*, 2021; Montgomery *et al.*, 2021; Reid *et al.*, 2022). Two studies employed mixed methods research designs (Baxter *et al*., 2024; Sharma, 2023) whereas all other studies used qualitative research methodologies, often through multi-phase approaches.

### Who is involved in the co-design process?

Interventions were co-designed with individuals with diverse lived experiences of marginalization, including unemployed young (22 years old on average) pregnant mothers (Abbass-Dick *et al.*, 2021), women survivors of childhood sexual abuse who were pregnant or wished to become parents (Montgomery *et al.*, 2021), parents with mental illnesses caring for young children (Goodyear *et al.*, 2021), new parents with low literacy levels (Muscat *et al.*, 2021), pregnant individuals from diverse ethnic backgrounds and low socioeconomic status (Sharma *et al.*, 2023), families experiencing food insecurity (Baxter *et al.*, 2024), Aboriginal and Torres Strait Islander women and parents (Reid *et al.*, 2022; Williams *et al.*, 2024), and parents experiencing social adversities such as socio-economic deprivation, maltreatment, or household/community dysfunction (Hall *et al.*, 2023). Two studies included only mothers (Abbass-Dick *et al.*, 2021; Montgomery et al., 2021) and four studies included both parents in the co-design process (Goodyear *et al.*, 2021; Muscat *et al.*, 2021; Reid *et al.*, 2022; Hall *et al.*, 2023), with numbers of participants ranging from 2 to 37 (Table 1).

All studies included health- and social-service providers caring for the structurally marginalized groups as co-design stakeholders with numbers ranging from 2 to 37 participants. Participants were identified based on strategic provider positions and targeted invitations to participate in the intervention co-design (Abbass-Dick *et al.*, 2021; Goodyear *et al.*, 2021; Reid *et al.*, 2022; Hall *et al*, 2023; Baxter *et al.*, 2024; Williams *et al.*, 2024). An invitation for participation was sent by an electronic flyer to health- and social-service providers in studies by Reid *et al*., (2022) and Hall *et al*., (2023). The latter mentioned an application requirement as part of the selection process. Some studies provided no (Montgomery *et al.*, 2021; Muscat *et al.*, 2021) or little information on the recruitment process of the health- and social-service providers (Abbass-Dick *et al.*, 2021; Goodyear *et al*., 2021; Reid *et al.*, 2022; Sharma *et al.*, 2023).

### How and when do structurally marginalized populations participate in co-design?

Table 3 summarizes the activities undertaken before, during, and after the co-design process, including activities proposed in the framework by Dietrich *et al.* (2017) on co-designing with “vulnerable” populations. Prior to undertaking the active co-design phase, four studies conducted a scoping review to better understand the local context and the evidence from existing interventions (Goodyear *et al.*, 2021; Reid *et al.*, 2022; Hall *et al.*, 2023; Baxter *et al.*, 2024). Three studies formed a project advisory group to establish governance and to oversee the co-design process, which included at least one representative with lived experiences of structural marginalization (Montgomery *et al.*, 2021; Hall *et al.*, 2023; Williams *et al.*, 2024). Several studies conducted consultations with health- and social-care service providers to assess the feasibility of the workforce’s capacities and infrastructures required to implement the desired intervention (Abbass-Dick *et al.*, 2021; Hall *et al.*, 2023; Sharma *et al.*, 2023; Baxter *et al.*, 2024; Williams *et al.*, 2024).

**Table 3.** Co-design activities undertaken before, during, and after intervention co-design

	Co-design activities	Abbass-Dick 2021	Baxter 2024	Goodyear 2022^*^	Hall 2023^*^	Montgomery 2021	Muscat 2021	Reid 2022	Sharma 2023	Williams 2024
**Prior** to co-design process	Experts do the initial research/resourcing			X	X		X	X	X	X
	Scoping review prior to intervention co-design process		X	X	X			X		
	Creation of a project advisory group				X	X				X
	Recruitment through established partnerships	X	X			X		X		X
	Users are prepared to engage in co-design				X			X		
**During** co-design process (types of contributions made by participants, methods of participants’ involvement & time commitment)	Experts participate as co-design facilitators				X			X	X	
	Suggestions sought prior to intervention design	X				X	X	X		
	Input sought during intervention design	X	X	X	X	X	X	X	X	X
	Workshop		X	X	X			X	X	X
	Focus group	X				X			X	X
	One on one interviews	X	X		X	X	X		X	
	Remote option available (e.g., phone interview, online survey)	X	X	X		X			X	X
	Number and estimated duration of meetings	2x unknown duration	3x unknown duration	6x unknown duration	7x full days	2x2h	1x unknown duration	1x3h	1x unknown duration	2x unknown duration
**After** co-design process	User-testing in the wider community	X	X		X	X	X		X	X
	Recognition of the expertise of end users in evaluation	X	X	X	X	X	X	X	X	X

* These studies contributed to the establishment of practice model.

In the active phase of intervention co-design, all but one study used a stepwise process (Muscat *et al.*, 2021). Across the studies, these phases generally included eliciting input from end-users regarding what the intervention should entail, followed by developing a first iteration of the intervention and then seeking feedback on this first iteration.

As shown in Table 3, in terms of stakeholder contributions, most studies involved seeking suggestions from participants prior to the development of the intervention/resource (Abbass-Dick *et al.*, 2021; Montgomery *et al.*, 2021; Muscat *et al.*, 2021; Reid *et al.*, 2022). Half of the studies asked participants to provide suggestions on various aspects of the services being co-designed (Abbass-Dick *et al.*, 2021; Muscat *et al.*, 2021; Reid *et al.*, 2022). For example, two studies asked participants what they would like to see in a future resource designed for them (Abbass-Dick *et al.,* 2021; Montgomery *et al.*2021). In the study by Montgomery *et al.* (2021), women were asked to share their hopes, fears, questions, and what they wished they had known before becoming pregnant. Some studies asked participants for feedback on a prototype, an existing resource or a revised resource (Abbass-Dick *et al.*, 2021; Montgomery *et al.*, 2021; Baxter *et al.*, 2024). For example, in Phase Two of the study by Abbass-Dick *et al.* (2021), mothers provided feedback on a revised eHealth tool designed based on the recommendations they had provided in a previous phase. Wider community user testing of the resource being co-designed was reported in seven studies (Abbass-Dick *et al.*, 2021; Montgomery *et al.,* 2021; Muscat *et al.*, 2021; Sharma *et al.*, 2023; Hall *et al.*, 2023; Baxter *et al.*, 2024; Williams *et al.*, 2024).

The methods used to gather input/feedback were described in all studies to some extent: three studies used both interviews and focus groups (Abbass-Dick *et al.*, 2021; Montgomery *et al.*, 2021; Sharma *et al.*, 2023), one study used interviews only (Muscat *et al.*, 2021), and six studies used group workshops (Goodyear *et al.*, 2021; Hall *et al.*, 2023; Reid *et al.*, 2022; Sharma *et al.*, 2023; Baxter *et al.*, 2024; Williams *et al.*, 2024). To increase participation, remote options were made available in six studies (online surveys and phone interviews) (Abbass-Dick *et al.*, 2021; Goodyear *et al.*2021; Montgomery *et al.*, 2021; Sharma *et al.*, 2023; Baxter *et al.*, 2024; Williams *et al.*, 2024). Two studies used a pre-existing tool for data collection and design (Abbass-Dick *et al*., 2021; Hall *et al.,* 2023). The tool for Abbass-Dick *et al.* (2021) comprised a structured table where participants could record their ideas, whereas Hall *et al.* (2023) used different tools, such as personas or storyboards. Hall *et al.* (2023) also reported the importance of considering multiple ways of knowing and participating to understand the issue that needs to be addressed through the creation of client persons who captured different community members’ lived experiences. They also provided various opportunities for other community members who were not directly involved in the co-design team but still wanted to contribute to the intervention (Hall *et al.,*2023).

The time commitment for participants was heterogeneous across studies and reported in limited detail overall. Time commitment varied from one time (approximately 3h) to 7 full days over a year. For example, Reid *et al.* (2022), held one three-hour in-person session. In other studies, participants could share their opinion twice over a short period of time (Abbass-Dick *et al.*, 2021; Montgomery *et al.*, 2021) or multiple times over one year (Goodyear *et al.*, 2021; Hall *et al.*, 2023). Two studies focused on the development of a model of care which required more investment by the participants (Goodyear *et al.*, 2021; Hall *et al.*, 2023). In the study by Goodyear *et al.* (2021), participants took part in six workshops during which they were asked to make key decisions about the service being developed.

### Which strategies are used to reach and engage structurally marginalized populations in the co-design process?

Most participants experiencing structural marginalization were recruited for the co-design process through an organization they were already a part of (e.g., the Canadian Prenatal Nutrition Program, the Survivor Trust, Healing the Past by Nurturing the Future Project) (Abbass-Dick *et al.*, 2021; Montgomery *et al.*, 2021; Reid *et al.*, 2022). In terms of the strategies used to facilitate participation in the co-design process, two studies provided financial compensation ranging from $25 to $100 (Abbass-Dick *et al.*, 2021; Montgomery *et al.*, 2021), and two studies did not report the compensation amount (Hall *et al.*, 2023; Sharma *et al.*, 2023). Two studies reimbursed transportation costs and childcare expenses (Montgomery *et al.*, 2021; Reid *et al.*, 2022). Another strategy mentioned to enhance engagement was promoting trust through transparent governance (Hall *et al.*, 2023). However, other than childcare provision and offering options for remote participation, specific strategies unique to prenatal, postpartum and early childhood periods were seldom discussed.

### What are practical and ethical considerations when co-designing health interventions with structurally marginalized populations?

Practical challenges were discussed in most studies (Abbass-Dick *et al.*, 2021; Goodyear *et al.*, 2021; Montgomery *et al.*, 2021; Reid *et al.*, 2022; Hall *et al.*, 2023). Low attendance at workshops or focus groups was mentioned in four studies (Abbass-Dick *et al.*, 2021; Goodyear *et al.*, 2021; Montgomery *et al.*, 2021; Hall *et al.*, 2023). For example, Hall *et al.* (2023) mentioned participants’ difficulty in juggling the time commitment requirements of the co-design project with other responsibilities in families with young children (e.g., caregiving, work). Participants in the study by Montgomery *et al.* (2021) reported difficulty in attending co-design activities in person. Another challenge reported in two studies was ensuring the representativeness of stakeholders (Reid *et al.,* 2022; Hall *et al.*, 2023; Baxter *et al.*, 2024). For example, Reid *et al.* (2022) questioned the representativeness of the stakeholders who attended the workshop versus the rich and varied experiences within the Aboriginal and Torres Strait Islander communities, especially in relation to parenting practices. Reid *et al.* (2022) also mentioned using decolonizing research methods and choosing the location of the workshop to be on traditional lands for the creation of a safe space. One study by Hall *et al.* (2023) reported the challenge of engaging with community members who did not speak English; this study did not use official interpreters in co-design activities. Lastly, Goodyear *et al.* (2021) reported the importance of organizational support and buy-in from health authorities to support the integration of the co-designed intervention into standard health- and social-service care.

In terms of ethical considerations, only one study mentioned the importance of negotiating power differentials with an iterative negotiation of power between the stakeholders and by ensuring the design process engaged everybody on equal footing (Hall *et al.*, 2023). No other ethical concerns were raised, and no studies discussed ethical considerations related to involving structurally marginalized populations in the prenatal and early childhood periods in co-design activities that requested significant time commitment.

## Discussion

This rapid scoping review highlights the scarcity of peer-reviewed published research on the co-design of health interventions and services with structurally marginalized populations in maternal and early childhood care. Only nine studies met the inclusion criteria, all published in the last four years. Most studies used a stepwise process to co-design interventions namely by eliciting input from end-users regarding what the intervention should entail, followed by developing a first iteration of the intervention and seeking feedback on this first iteration. To reach and engage structurally marginalized populations, participants in the co-design process were mainly recruited through existing community organizations. Ethical and practical considerations stemming from co-designing with structurally marginalized populations during prenatal and early childhood periods were often not addressed or addressed only superficially. Although contributions on specific aspects of co-design in the maternal and early childhood context are limited, this review improves our overall understanding of the co-design process with structurally marginalized populations.

The scarcity of published studies raises the question of whether co-design with structurally marginalized populations during prenatal and early childhood periods is an emerging concept, is underreported in the scientific literature, or faces other barriers that make it more difficult to reach and engage structurally marginalized populations (e.g., language barriers, caregiving responsibilities). Compared to review studies on co-design with structurally marginalized populations not focused on prenatal/early childhood periods by King *et al.* (2022), Rogers *et al.* (2020), Rustage *et al*. (2021) and Samb *et al*. (2019), which included between 15 and 28 studies, this study only included nine studies focused on the intersection of structural marginalization and maternal and child health.

The co-design process of each study included in this review was unique, especially with regards to recruitment approaches and types of contributions made by stakeholders, which reinforces the importance of adaptation to the context in which co-design is taking place. The need to stay flexible in terms of time and meeting location was reported throughout studies included in our review, which is in accordance with the findings of Amann and Sleigh (2021) and Mulvale *et al.* (2019). The use of community intermediaries and multiple methods to gather contributions, as well as the importance of ensuring adequate resources for the full co-design process has been mentioned previously as strategies to optimize co-design of health services with ethnic minority consumers (Chauhan *et al*. 2021).

The studies included in our review provide limited insight on co-designing with populations in the prenatal and early childhood period. Most studies did not mention specific adaptations or co-design implementation approaches to address the unique needs of individuals and families expecting or caring for a young child (e.g., provision of childcare or other financial incentives). Others have commented on such considerations, albeit not in the context of structural marginalization. For example, specific considerations reported in a study on the co-design of a lactation mHealth tool with breastfeeding mothers included anticipating that little time is available from mothers and that children who might be present during meetings can be a source of distraction during co-design activities (Wardle *et al.*, 2018). Being flexible with the data-gathering tools (e.g., providing access to an online survey instead of participating in a group discussion), meeting places, and scheduling were reported as important factors when co-designing in maternal child health contexts (Wardle *et al.*, 2018). Similarly, providing incentives, being empathic with co-design partners, and connecting with participants from the perspective of a lived experience may yield positive participation results (Walker *et al*., 2020, 2018). These factors may be even more important when co-designing with populations at the intersection of prenatal/early childhood and structural marginalization.

Despite the need for flexibility in the co-design process, using standardized co-design methods and reporting detailed co-design protocols can contribute to ensuring research quality, replicability of findings, and safety of structurally marginalized stakeholders (O’Brien and Fossey, 2021). None of the studies included in our review mentioned using a framework specific to co-design or to structurally marginalized populations. We used a co-design with “vulnerable” consumers framework to synthesize steps in the co-design processes of included studies (Dietrich *et al*. (2017). However, this framework was difficult to apply to health-related interventions due to the framework’s reliance on language and definitions that lacked clarity. Therefore, a framework tailored specifically for health care interventions is needed, which incorporates flexibility in its application and provides detailed descriptions and definitions for each step of the co-design process.

Among the challenges identified across the studies we reviewed, several were similar to those identified by Mulvale *et al.* (2019), namely competing demands, significant time commitment, high attrition rates, and lack of representativeness. However, no studies raised concerns about the potential stigma, emotional labor, or distress that may be experienced by structurally marginalized parents and families as a result of participating in co-design activities. Meeting the stakeholders’ needs during the co-design of health interventions such as providing emotional, financial, and psychological support and ensuring confidentiality, was not addressed. More attention is needed on these topics, particularly in co-design with structurally marginalized populations for maternal and early childhood care (Mulvale *et al.*, 2019). The importance of addressing power imbalances prior to engaging structurally marginalized populations was reported by some studies, and the inclusion of end-users in the decision-making and in the governance of co-design projects was used as a strategy to reduce potential power differentials (Mulvale *et al.,* 2019; Chauhan *et al.,* 2021; Butler *et al.,* 2022). This review highlights the importance of implementing approaches and strategies to minimize participation burden and include benefits for participants who already face multiple intersecting challenges, and for whom participating in research may be of low priority.

### Strengths and limitations of the study

This scoping review can inform future co-design approaches with populations in prenatal and early childhood contexts among populations with experiences of structural marginalization. Although this review is not the first to explore co-designed interventions with end-users facing contexts of vulnerability (Samb *et al*., 2019; Rogers *et al.*, 2020; Rustage *et al*., 2021; King *et al.*, 2022), it is the first to focus specifically on prenatal and early childhood periods, periods of potentially added vulnerability for individuals and families. Another strength of this knowledge synthesis is the rigorous methodological approach that was used. A systematic process guided by the JBI guidelines (Peters *et al.,* 20015, 2020) and the PRISMA-ScR reporting guidelines (Tricco *et al.,* 2018) was followed to increase the validity and reproducibility of findings from this rapid scoping review. Nevertheless, co-design is a relatively new concept that lacks a clear definition which may have led to the omission of studies that used definitions and terms not included in our search strategy. To mitigate this risk, our search was as broad as possible, encompassing co-design, co-production, and co-creation as search terms. Furthermore, the exclusion of gray literature may have resulted in the omission of reports on health intervention co-design that fall outside the academic realm. Given that this review focused on structurally marginalized populations, these groups may be less likely to publish in academic peer-reviewed journals. In addition, among included studies, the description of co-design approaches was sometimes brief with limited details provided, which may have led to an incomplete description of approaches in the current review. Lastly, we did not appraise the quality of the studies reviewed. Although this is an optional step in scoping reviews, doing so strengthens the synthesized evidence.

### Implications and recommendations for practice and research

Researchers and health- and social-care service providers interested in co-designing health interventions by engaging with structurally marginalized populations should understand that co-design is a broad and superficially defined term. Although there is diversity in how co-design is being done and who is involved, engagement with individuals/families and other stakeholders has generally been done in three phases (i.e., pre-co-design, active co-design, and post-co-design). Future research should aim to clarify the concept of co-design and develop a functioning definition and a standardized yet flexible framework to guide future co-design of health interventions involving structurally marginalized populations. Moreover, future research on co-designing prenatal and early childhood interventions should better describe considerations and measures implemented to meet the needs of this unique population and avoid unintended consequences.

Due to the specific challenges arising from engaging with marginalized populations, critical reflections about the population intended to engage in co-design should be conducted prior to the process to prevent futile and performative co-design. Researchers should question their privileges and identify the power dynamics that may be present between the stakeholder groups. To reduce any power differential, equal opportunities should be given to all participants to contribute not only to the co-design process but also in the governance of the project.

Lastly, similarly to Amann and Sleigh (2021) and Mulvale *et al.* (2019), we recommend the establishment of communities of practice to share knowledge and unify the co-design process for members of different population groups that experience vulnerability factors including structural marginalization. This could be done through a virtual hub where resources and experiences can be exchanged. Moreover, to standardize the reporting of the co-design process, we recommend that future studies better report the steps and activities conducted in the co-design process. Using guidelines such as GRIPP2 for reporting on patient and public involvement in health- and social-care research could help to ensure that the research focuses on issues relevant to patients and the public by increasing transparency (Staniszewska *et al.*, 2017).

## Conclusion

This rapid scoping review synthesizes evidence on co-design approaches with structurally marginalized populations in primary maternal and early childhood care. Findings from this study can support organizations that provide health and social services to these populations and are interested in co-designing interventions and services within their settings. Organizations interested in co-designing with structurally marginalized populations during the maternal and early childhood context should engage in critical reflections on the power differentials and understand the need for flexibility to support positive engagement. Using co-design with structurally marginalized populations can result in greater acceptability of the resource being developed; however, some challenges remain to ensure that their involvement does not add any additional burden.

## Supporting information

Vicat-Blanc et al. supplementary material 1 Vicat-Blanc et al. supplementary material

Vicat-Blanc et al. supplementary material 2 Vicat-Blanc et al. supplementary material

## Supplementary material

The supplementary material for this article can be found at https://doi.org/10.1017/S146342362510011X.
