# Supplementary material for: Co-design of interventions and services with structurally marginalized populations in the context of maternal and early childhood primary care: a rapid scoping review
Source: Prim Health Care Res Dev. 2025 Jun 16;26:e48. doi: 10.1017/S146342362510011X (PMC12175099; doi:10.1017/S146342362510011X)
Supplement: Vicat-Blanc et al. supplementary material 2 — Vicat-Blanc et al. supplementary material [file S146342362510011Xsup002.pdf]

Vicat-Blanc et al. Co-design of interventions and services with structurally marginalized populations in the context of maternal and early childhood primary care: a rapid scoping review

Supplemental File 2

Search Strategy (Embase)

	Structurally marginalized	AND	Co-design
	(marginali*.tw,kf		Co-design. Tw,kf
	Disparit* adj3 (socioeconomic or health).tw.kf		codesign. Tw,kf
	((ethnic or minority) adj3 group*) .tw,kf		(co-produc* or coproduc*). Tw,kf
	((exclusion or isolation or welfare) adj2 social*).tw,kf		(co-creat* or cocreat*). Tw,kf
	Intimate partner violence.tw,kf		(co-construct* or coconstruct*). Tw,kf
OR	Racial*.tw,kf		Knowledge mobili*. Tw,kf
	Racis*. tw,kf		
	Race*. tw,kf		
	Ethnic*. tw,kf		
	Cultur*. tw,kf		
	Religio*. tw,kf		
	Migra*. tw,kf		
	Immigra*. tw,kf		
	Refugee*. Tw,kf		
	Disab*. Tw,kf		
	Gender*. Tw,kf		
	Sexua* . Tw,kf		
	LGBT. Tw,kf		
	Housing/		
	Exp housing instability/		
	Exp Food insecurity/		
	Exp domestic violence		
	Exp minority group/		
	Exp ethnic group/		
	Exp vulnerable population/		
	Exp health disparity/		
	Race difference/		
	Exp migrant/		
	Social isolation/		
	Exp social exclusion/		

Search Strategy (CINAHL)

	Structurally marginalized	AND	Co-design
	TX (exclusion or isolation or welfare) N2 social*		"Knowledge mobili*"
	TX Disparit* N3 (socioeconomic or health or race)		"coprod*"
	"marginali*"		"co-prod*"
	TX (ethnic or minority) N3 group*		"cocreat*"
	TX religio*		"co-creat*"
OR	TX cultur*		"co-design"
	TX ethnic*		"codesign"
	TX racial*		"co-construct*"
	TX racis*		"coconstruct*"
	MH "Intimate Partner Violence"		
	TX intimate partner violence		
	TX religio*		
	TX LGBT*		
	TX sexua*		
	TX gender*		
	TX disab*		
	TX refugee*		
	TX immigra*		
	MH "Social Isolation+"		
	MH "Residential Mobility+"		
	MH "Race Relations+"		
	MH "Ethnic Groups+"		
	MH "Minority Groups"		
	MH "Domestic Violence+"		
	MH "Food Security+"		
	MH "Health Status Disparities+"		
	MH "Health Knowledge"		
	MH "Health Literacy"		
	MH "Health Care Delivery, Integrated"		
	MH "Healthcare Disparities"		
	MH "Health Inequities"		
